# Ancient Transcription Factors in the News

**DOI:** 10.1128/mBio.01547-18

**Published:** 2019-02-26

**Authors:** Irina Artsimovitch, Stefan H. Knauer

**Affiliations:** a Department of Microbiology and The Center for RNA Biology, The Ohio State University, Columbus, Ohio, USA; b Lehrstuhl Biopolymere, Universität Bayreuth, Bayreuth, Germany; University of Texas Health Science Center at Houston

**Keywords:** NusG, RfaH, antitermination, transcription, translation

## Abstract

In every cell from bacteria to mammals, NusG-like proteins bind transcribing RNA polymerase to modulate the rate of nascent RNA synthesis and to coordinate it with numerous cotranscriptional processes that ultimately determine the transcript fate. Housekeeping NusG factors regulate expression of the bulk of the genome, whereas their highly specialized paralogs control just a few targets. In Escherichia coli, NusG stimulates silencing of horizontally acquired genes, while its paralog RfaH counters NusG action by activating a subset of these genes. Acting alone or as part of regulatory complexes, NusG factors can promote uninterrupted RNA synthesis, bring about transcription pausing or premature termination, modulate RNA processing, and facilitate translation. Recent structural and mechanistic studies of NusG homologs from all domains of life reveal molecular details of multifaceted interactions that underpin their unexpectedly diverse regulatory roles. NusG proteins share conserved binding sites on RNA polymerase and many effects on the transcription elongation complex but differ in their mechanisms of recruitment, interactions with nucleic acids and secondary partners, and regulatory outcomes. Strikingly, some can alternate between autoinhibited and activated states that possess dramatically different secondary structures to achieve exquisite target specificity.

## Abstract (precis)

In every cell from bacteria to mammals, NusG-like proteins bind transcribing RNA polymerase to modulate the rate of nascent RNA synthesis and to coordinate it with numerous cotranscriptional processes that ultimately determine the transcript fate. Housekeeping NusG factors regulate expression of the bulk of the genome, whereas their highly specialized paralogs control just a few targets.

## INTRODUCTION

All cellular genomes are transcribed by multisubunit, evolutionarily related RNA polymerases (RNAPs), whose function is elaborately regulated by a plethora of divergent accessory factors (1). Among those regulators, NusG homologs stand out as the only universally conserved family of transcription factors (2). These proteins exert a combination of direct and indirect effects on gene expression through modifying the properties of transcription elongation complexes (TECs) and bridging RNAP to diverse proteins involved in RNA processing, modification, translation, etc. In addition to ubiquitous housekeeping NusG proteins (called Spt5 in archaea and eukaryotes and DRB-sensitivity-inducing factor [DSIF] in humans), which associate with RNAP transcribing most of the cellular genome (3, 4), many species also encode specialized NusG paralogs (NusG^SP^), which modulate expression of a subset of genes, sometimes acting orthogonally to the essential (in most cases) primary NusG (5).

Escherichia coli NusG, the founding member of this family, has been identified along with other N-utilization substances (Nus) proteins as a cellular factor required for bacteriophage λN-mediated antitermination within delayed early genes (6, 7). This antitermination activity is orthogonal to the main cellular role of NusG, i.e., to aid the termination factor Rho in silencing harmful horizontally acquired DNA in E. coli (8). The first specialized NusG paralog, RfaH (also known as SfrB and HlyT), was first identified in E. coli (9) and *Salmonella* (10). RfaH opposes the Rho-stimulating action of NusG to activate expression of some xenogenes (11, 12). Biochemical, genetic, and structural studies of E. coli NusG and RfaH contributed the bulk of information about the molecular mechanisms and regulatory diversity of this class of proteins with important insights from studies of NusG homologs from diverse phyla.

While this family of regulators has been studied since the early 1980s, recent advances in whole-genome and structural analyses are rapidly reshaping our views and opening new areas of investigation. In this minireview, we will focus on recent findings, and we direct the reader to more comprehensive reviews that do more justice to the history of this fascinating family (2, 5, 13, 14). In particular, we will cover new insights from structural studies of NusG proteins, revealing molecular details of interactions with RNAP, other accessory factors, and nucleic acids that determine their effects on transcription. We will concentrate on bacterial regulators, drawing parallels with their eukaryotic homologs to highlight the universal principles of RNA synthesis control. We sincerely apologize for failing to cite work of many colleagues whose contributions have defined the field and made recent advances possible.

### NusG and RfaH: similarities and differences.

**FIG 1:**
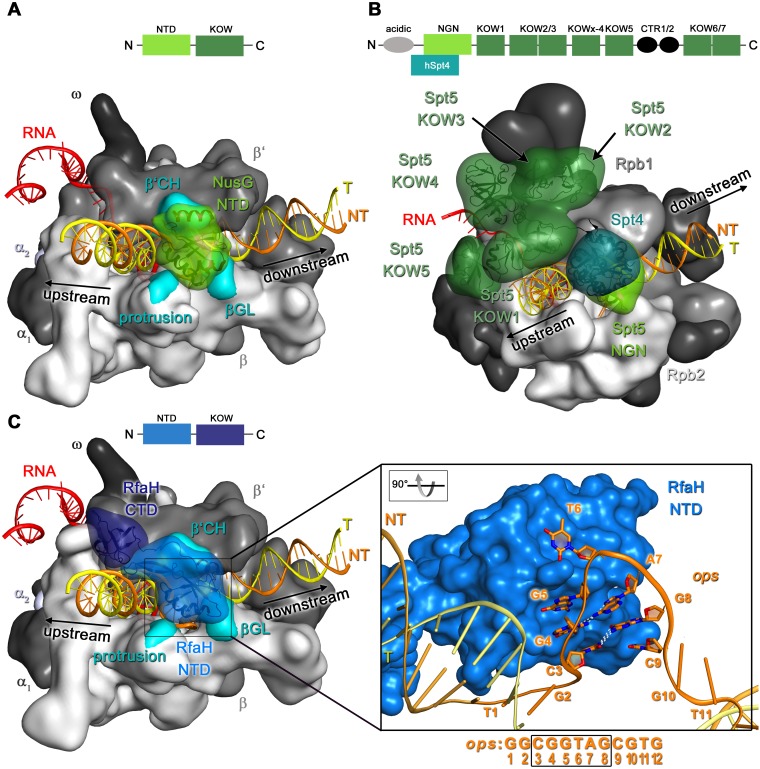
Structures of elongation complexes with bound NusG proteins. The schematics above each panel indicate the domain arrangement in the respective NusG protein. RNAP is in surface representation, NusG is in surface and ribbon representation, and nucleic acids are in ribbon representation. (A) E. coli TEC with NusG (PDB ID 6C6U). NusG-NTD contact surfaces are highlighted in cyan. (B) Mammalian RNAP II TEC with DSIF (PDB ID 5OIK). (C) E. coli TEC with RfaH (PDB ID 6C6T), with NTD contacts shown as in panel A. The inset shows an enlargement of the boxed region. The central *ops* nucleobases are depicted as sticks with N atoms in blue and O atoms in red. White dashed lines indicate hydrogen bonds between base pairs C3:G8 and G4:A7 that form the stem of the *ops* hairpin. The *ops* sequence is shown below with the central bases essential for RfaH recruitment boxed.

Extensive structural and functional data available for E. coli RfaH and NusG reveal a mix of common and divergent properties (Table 1). The two proteins bind to RNAP roughly in the same place, as do all other NusG homologs (Fig. 1), but their contacts to the enzyme are not identical. RfaH binds to the transcribing RNAP more tightly (15), as is required to fend off competition with the more abundant NusG (11), and recognizes a specific DNA sequence during recruitment (11). NusG appears to be recruited to RNAP at random sequences (4) and does not interact with DNA in the structure of a NusG-bound scaffold TEC (15); however, it is possible that, similarly to its Bacillus subtilis ortholog (16), E. coli NusG has some nucleic acid sequence preferences. NusG and RfaH also differ in their effects on transcription. While both factors counteract backtracking (17–20), only RfaH can suppress the effects of pause-stabilizing effects of nascent RNA hairpins (21–23). Neither protein exhibits strong effects at intrinsic terminators, although both factors have been reported to decrease termination 2-fold at selected sites *in vitro* (24, 25); antitermination of an intergenic *hly* terminator observed *in vivo* by RfaH is thought to contribute to the activation of hemolysin expression (26). NusG and RfaH make similar contacts to ribosomal protein S10 (also known as NusE) as observed by nuclear magnetic resonance (NMR) spectroscopy (27, 28); these contacts are proposed to bridge RNAP and the ribosome (29) and to mediate 30S recruitment by RfaH (27).

**TABLE 1:** Summary of main properties of E. coli RfaH and NusG^*b*^

Property, interaction, and/or activity	NusG	RfaH
Interdomain contacts in free protein	None	Extensive
CTD fold	β-barrel	α-Helix in free protein β-barrel when bound to TEC
Sequence requirement for recruitment to RNAP	None	*ops* element
Contact sites on the β′ subunit	Clamp domain, CH	Clamp domain, CH
Contact sites on the β subunit	GL, lobe, protrusion	GL, lobe, protrusion,^*a*^ flap
Interactions with the nontemplate DNA strand	Not observed	Yes, stabilizes a DNA hairpin
Interactions with NusE (S10)	Yes	Yes
Interactions with Rho	Yes	No
Effect on the upstream edge of the transcription bubble	Stabilizes	Stabilizes
Effect on backtracking	Inhibits	Inhibits
Effect on hairpin-stabilized pausing	None	Inhibits
Effect on Rho-dependent termination	Activates	Inhibits
Effect on translation	Proposed to couple RNAP and 70S during elongation	Proposed to recruit 30S during initiation

a In the *ops*-TEC, RfaH contacts with the protrusion are blocked by the NT-DNA hairpin. Once RNAP escapes from the *ops* site, RfaH is expected to make contacts to the protrusion, as observed for NusG.

b CH, clamp helices; GL, gate loop.

The defining functional difference between RfaH and NusG lies in their opposite effects on Rho-dependent termination. NusG stimulation of Rho activity is evident *in vitro* (30, 31), even in the absence of RNAP; NusG directly binds to Rho and facilitates its isomerization into a closed, active state (32). RfaH does not interact with Rho under physiological conditions and has only a mild antitermination effect *in vitro* (22), in sharp contrast to potent antitermination observed *in vivo* (12). These results argue that RfaH inhibits Rho indirectly by excluding NusG and facilitating the ribosome loading onto mRNA (11, 27).

### Structural basis for antipausing.

As the only ubiquitous family of transcription factors, NusG-like proteins are expected to share some modes of action. Indeed, despite their significant sequence divergence, NusG/Spt5 interactions with and effects on the TEC are broadly conserved (15, 33–35). Most functionally characterized NusG homologs reduce transcriptional pausing and arrest, thereby favoring productive RNA synthesis, through conserved interactions with RNAP (17, 20, 36, 37). However, this ability can be masked at regulatory sites (38), as sequence-specific contacts to DNA by E. coli RfaH (22) and B. subtilis NusG (16, 39) hinder RNAP escape.

NusG-like proteins have a modular structure and share a similar core architecture (Fig. 1), consisting of at least two flexibly tethered domains, an N-terminal domain (NTD; also known as NGN, NusG N-terminal domain) with conserved, mixed α/β topology (40), and a C-terminal domain (CTD) that contains a Kyrpides, Ouzounis, Woese (KOW) motif (41) and folds into a five-stranded antiparallel β-barrel. Bacterial NusGs exhibit this basic two-domain structure (Fig. 1A), but some have additional domains inserted into the NTD (42). Archaeal Spt5 proteins are also composed of an NTD and a single KOW domain but form heterodimers with a small accessory factor Spt4. Increased regulatory complexity in eukaryotes is reflected by an even more elaborate structure of NusG homologs: Spt5 harbors an unordered acidic N terminus, the NGN domain, several KOW domains, and a mobile C-terminal repeat (CTR) region (Fig. 1B).

Within the last 2 years, many structures of active and paused TECs in the absence and presence of NusG/Spt5 proteins were obtained by cryo-electron microscopy (cryo-EM) and X-ray crystallography (15, 33, 34, 36, 38, 43–46). These structures, obtained with TECs ranging from bacteria to mammals, revealed common molecular principles for the regulation of elongation and pausing. RNAP is shaped like a crab claw with the two largest subunits (β′ and β in bacteria, Rpo1 and Rpo2 in archaea, and Rpb1 and Rpb2 in eukaryotic RNAPII) constituting the two pincers (47). The cleft formed by the pincers harbors the nucleic acid chains and contains the active site. In E. coli TEC, the downstream duplex DNA enters the active site cleft and separates at position +1 to place the template (T) strand +1 base into the active site, where it can pair with an incoming substrate NTP (45). The template DNA (T-DNA) pairs with the RNA to form a 9-bp hybrid, whereas the single-stranded nontemplate (NT) DNA is solvent accessible and flexible and not visible in most structures unless constrained by transcription factors (44–46). Conserved RNAP elements at the upstream edge of the RNA:DNA hybrid direct RNA and the T-DNA away from each other, preventing the formation of an extended DNA:RNA hybrid (45). The T-DNA reanneals with the NT-DNA, resulting in a distorted −10 bp, but leaving no single-stranded gap in the T-DNA strand (18, 45). The upstream DNA duplex is mobile and has only few interactions with RNAP, forming an ∼110° angle with the downstream DNA duplex (45).

The cryo-EM structures of E. coli TEC bound to NusG and RfaH (15) reveal the details of their interactions with RNAP and suggest several mechanisms of pause suppression. The NTDs bind to RNAP at similar positions, whereas the flexibly connected CTD is visible only in a subpopulation of the RfaH:TEC particles (Fig. 1A and C). The NTD is located at the upstream side of the clamp, contacting the clamp helices (CH) of the β′ pincer and the protrusion and gate loop (GL) of the β pincer, thus bridging the active site cleft and locking the nucleic acids inside. Functional studies implicated the β′CH region as a high-affinity binding site of RfaH and NusG and demonstrated that the NTD is sufficient for their antipausing effects (48, 49). The β′GL element, in contrast, is largely dispensable for binding and activity of NusG (18, 50) but contributes to the antipausing activity of RfaH (12). The NTD exerts several effects on the TEC structure. First, the NTD alters the path of the upstream DNA duplex without making any interactions with this DNA region. This effect is mediated by looping the NT-DNA strand out, which brings the upstream and downstream DNA duplexes closer together, and is particularly pronounced with RfaH. By changing the upstream DNA trajectory, the NTD indirectly stabilizes the −10 bp at the upstream fork junction of the transcription bubble. The −10 bp, which must melt during RNAP backtracking, is distorted in factor-free TECs (45), thus favoring backtracking. Stabilization of the −10 bp by RfaH and NusG, which was observed in cryo-EM structures (15) and confirmed by cross-linking (15, 18), provides a straightforward explanation for antibacktracking effects of the NTD. Second, the NTD may stabilize the active TEC state by sterically disfavoring subtle conformational changes (termed swiveling) observed in cryo-EM studies of hairpin-stabilized paused TECs (43, 44). This proposed antiswiveling effect was experimentally demonstrated only with the RfaH-NTD (15), consistent with the lack of NusG effects at hairpin sites. Third, the NTD is positioned to interact with the NT-DNA. No density of this NT-DNA segment was observable in the TEC:NusG structure (15), in agreement with the lack of apparent sequence specificity of E. coli NusG (4). In contrast, RfaH contacts the NT-DNA strand, which contains the RfaH recognition sequence (Fig. 1C; see below). Underscoring the ubiquity of these regulatory mechanisms, structures of eukaryotic TECs bound to DSIF and Spt4:Spt5 (33, 34) reveal that the Spt5-NGNs make similar bridging contacts at the cleft between the RNAP pincers to stabilize the bubble, interact with the upstream DNA duplex, and contact the NT-DNA (Fig. 1B). By constraining the path of the NT-DNA, NusG and Spt5 have been proposed to prevent it from assuming nonproductive conformations (50, 51); in support of this model, antipausing effect can be achieved by artificially shortening the NT-DNA strand in the TEC (52).

### NT-DNA interactions.

In the TEC, the central nucleotides of the single-stranded NT-DNA are solvent accessible and could thus be contacted by NTDs. Specific NT-DNA readout has been documented for B. subtilis NusG (16) and E. coli RfaH (22). In the latter case, recruitment to RNAP requires a conserved 12-nt operon polarity suppressor (*ops*) DNA element (14), located in untranslated leader regions of RfaH-activated operons. RfaH readily binds to the *ops* element in the NT-DNA in static TECs (22), but efficient recruitment to rapidly transcribing RNAP could be more challenging, particularly because RfaH cellular levels are low (53). Fittingly, in addition to making specific contacts to RfaH, the *ops* element halts RNAP to await RfaH arrival. *ops*, a backtrack-stabilized (class II) pause signal (21) that matches the consensus pause sequence, is the strongest pause in E. coli (54, 55). Functional analyses suggest that the consensus pause comprises a group of chimeric elements, of which *ops* is just one example; these signals could decelerate transcription and mediate specific interaction with diverse regulatory factors (56). The flanking, conserved regions of the pause element slow RNAP down, thus favoring the recruitment of regulators in low abundance kinetically. In contrast, the central region is short and variable, and both its primary and secondary structures must be read out by regulators to ensure tight control of recruitment (56). For example, RfaH is faithfully recruited to a few *ops* operons in E. coli while being vastly outnumbered by NusG (11).

To visualize the molecular details of DNA recognition by RfaH, the *ops*-paused TEC was used to obtain cryo-EM structures of RfaH and NusG complexes (15). While both RfaH and NusG are known to be active on the *ops*-TEC (21, 22), it represents a unique recruitment target for RfaH. These structures revealed striking differences between DNA conformations: the NT-DNA strand is invisible in NusG-bound TEC but forms a short hairpin that is recognized and stabilized by the RfaH-NTD (Fig. 1C). Structural and functional data demonstrate that the hairpin also forms in a binary RfaH:*ops* DNA complex and is essential for RfaH function (56). The stem of the hairpin is formed by two base pairs, a Watson-Crick C3:G8 base pair and a Saenger type XI G4:A7 base pair. In the loop region, a conserved T6 base is flipped out to fit into a binding pocket of RfaH-NTD, while G5 stacks on the upstream face of G4 and packs against the surface of RfaH-NTD (15, 56). Very few RfaH side chains form base-specific hydrogen bonds with DNA (15, 56), and each of these was shown to be important for RfaH function (57). Thus, binding specificity of RfaH is conferred by only a few direct interactions that are mediated by a secondary structure in the DNA. In contrast to other proteins that utilize flipping of a base to allow sequence-specific readout (58–63), RfaH does not use a wedge residue to mimic the flipped-out base. Instead, the NT-DNA hairpin exposes the two central *ops* nucleotides for specific recognition (15, 56), which is an alternative way of stabilizing a DNA conformation with a flipped-out base. The combination of conformational plasticity of the NT-DNA strand and neighboring RNAP features leads to rich regulatory diversity, allowing context-dependent recruitment of auxiliary factors in all domains of life.

A question of a “postrecruitment” conformation of RfaH-bound TEC following RNAP escape from the *ops* site remains to be addressed. Upon the loss of specific NT-DNA contacts that preclude interactions with the β protrusion domain, RfaH could form more extensive interactions with RNAP, explaining in part why it binds the TEC more tightly than NusG (15). By bringing the DNA duplexes closer together, the RfaH-NTD is expected to stabilize the NT-DNA strand in a looped-out, elongation-promoting conformation without making direct contacts to the DNA, which would likely hinder rapid RNA synthesis.

### Structural and regulatory diversity of NusG/Spt5-CTDs.

In contrast to the nearly identical interactions of NusG/Spt5-NTDs with the TEC that underlie their similar (except at sequences that make specific contacts) effects on transcription elongation, the CTDs mediate different interactions with other proteins that ultimately determine the regulatory effect of each NusG homolog. CTDs may also establish additional context-dependent contacts with the nucleic acids in the TEC (33, 34). For example, the KOW1 domain and the adjacent linker (L1) of Spt5 contact and guide the upstream DNA duplex in the TEC, whereas the KOW5 and KOWx-4 domains encircle the nascent RNA (Fig. 1B). These DNA and RNA clamps likely stabilize the TEC and increase its processivity and may also inhibit formation of R-loops in the wake of transcribing RNAP. Interestingly, in a subpopulation of RfaH:*ops-*TEC particles, the RfaH-CTD was bound on the RNAP surface near the RNA exit channel (15) at a location similar to that occupied by Spt5-KOW1 (33, 34). This observation suggests that the RfaH-CTD may alter the paths of the upstream DNA and the nascent RNA, thereby contributing to inhibition of hairpin-stabilized pausing and intrinsic termination (22, 25, 64).

**FIG 2:**
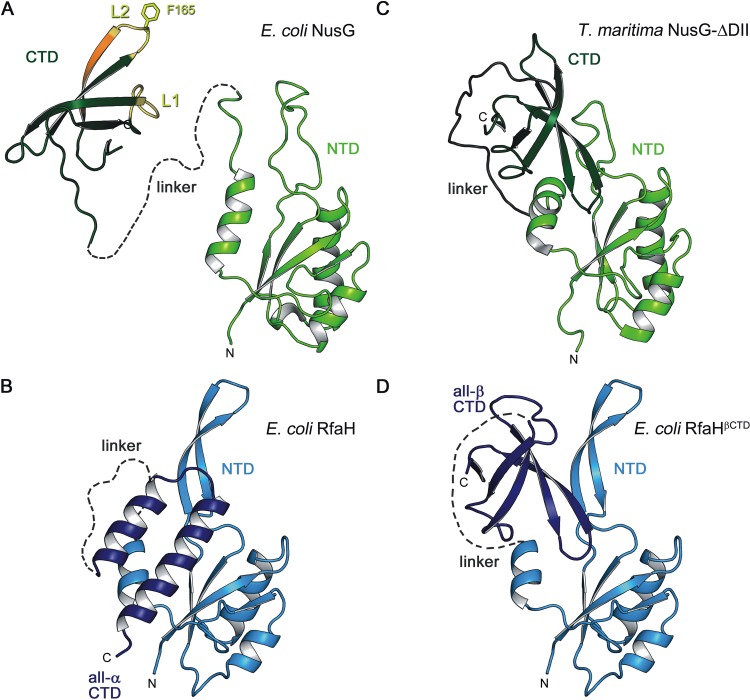
Domain arrangements of NusG and RfaH. Structures of E. coli NusG (A); E. coli RfaH (B); T. maritima NusG-ΔDII, a variant of T. maritima NusG where the additional domain DII is deleted (C); and E. coli RfaH, modeled in a closed state according to T. maritima NusG-ΔDII with its CTD in the all-β state (D). All structures are in ribbon representation. In panel A, the loops L1 and L2 of NusG-CTD that interact with Rho are highlighted in yellow. The region that is additionally involved in the NusG-CTD:S10 interaction is colored in orange. Phe165, which is essential for both NusG-CTD:Rho and NusG-CTD:S10 interaction, is depicted as sticks. PDB IDs: E. coli NusG-NTD, 2K06; E. coli NusG-CTD, 2JVV; RfaH, 5OND; RfaH-CTD all-β, 2LCL; T. maritima NusG, 2LQ8.

While most regulatory diversity of the CTDs is achieved through different contacts established by similarly folded β-barrel CTDs connected to the NTD by flexible linkers (Fig. 2A), the structure of free RfaH (48) revealed that its CTD was folded as an α-helical hairpin (Fig. 2B). In this autoinhibited state, the α-helical CTD tightly binds to and masks the β′CH binding site on the NTD, ensuring that RfaH does not bind to RNAP unless activated by the *ops* element. Upon recruitment, the domains dissociate and the CTD spontaneously refolds into a NusG-type β-barrel (27, 65).

### Autoinhibition as a regulatory mechanism.

Autoinhibition provides an elegant solution to a key regulatory challenge: how to direct NusG and RfaH, which bind to the same site on the TEC, to different genes? Since RfaH opposes the essential Rho-promoting NusG activity, its action must be narrowly confined to a few targets. While *rfaH* is not essential in lab-grown E. coli, its absence increases sensitivity to detergents, antibiotics, and bile (66, 67); reduces conjugation (68); increases biofilm formation (69); and attenuates virulence (70, 71). Optimal fitness thus depends on a balanced action of NusG and RfaH working alongside in the same cell. Their peaceful coexistence is made possible by marked differences in their recruitment strategies. A conventional σ-like binding to distinct sequence motifs is likely not feasible for NusG, which is associated with RNAP transcribing most of the genome (4) but appears to be bound only loosely (15), necessitating frequent rebinding. In contrast, RfaH activates just a few targets, making recruitment to a specific site a viable strategy.

Quite unusually, RfaH recruitment requires not only base-specific contacts between RfaH-NTD and *ops* but also domain dissociation to expose the high-affinity β′CH-binding site on the RfaH-NTD (Fig. 2B). The relief of autoinhibition is achieved when RfaH binds to RNAP paused at the *ops* site and is thought to be triggered by the recognition of the *ops* DNA hairpin and some RNAP element, most likely the βGL (65). While a short DNA oligomer containing *ops* can bind to RfaH (at high concentrations) and establish contacts that are similar to those observed in the complete *ops*-TEC, RfaH remains in the autoinhibited state in the binary complex (56, 65).

The available data argue that the stability of the interdomain interface determines the maintenance of the alternative α-helical RfaH-CTD fold and, thus, autoinhibition, since isolated RfaH-CTD spontaneously folds into the β-barrel conformation (27, 48, 72). Analysis of the autoinhibited RfaH structure identified several residues predicted to be critical for the domain interactions. Phylogenetic analysis of the NusG family suggested that among these residues, RfaH I93 and F130 could be essential for the unique properties of RfaH: these residues are highly conserved among RfaH orthologs but are different, and equally conserved, among NusGs. Consistent with this prediction, substitution of either residue for its NusG counterpart (I93E and F130V) converted RfaH into a NusG-like regulator that lost dependence on *ops* even though neither residue is involved in direct interactions with *ops* (73). Molecular dynamics simulations performed by several groups using different methodologies identified multiple candidate mechanisms for the α→β conversion of the RfaH-CTD but were all in agreement on the central role of F130 in this conversion (74–77). One study also highlighted the contribution of I93 therein (77).

In cells that encode more than one NusG homolog, differential targeting should be enabled to insulate the NusG regulon from interference, but no information is available on the mechanism of recruitment of any NusG^SP^ other than RfaH. We argue that the acquisition of autoinhibition may represent a relatively late step in the evolution of RfaH. In relatively recent NusG duplication events, targeted recruitment could be achieved in *cis*, a model consistent with observations that many NusG^SP^s are encoded within or near the operons they control (11). The availability of structural information on both sets of interactions and sequences of numerous NusG homologs should enable us to start addressing this question. By combining ancestral reconstructions of the NusG^SP^ family with the biochemical and structural analysis of the putative key intermediates, we expect to trace the evolution of this universally conserved family of transcription factors. We note that autoinhibition does not have to be coupled to the CTD transformation. We showed that in Thermotoga maritima NusG, interdomain interactions between the NTD and the β-barrel CTD mask the binding sites for Rho, S10, and RNAP and must be broken to achieve activation (Fig. 2C). In this case, the autoinhibited state is argued to stabilize the protein, a function that may be important in the hyperthermophilic niche of T. maritima (42).

Spontaneous refolding of the RfaH-CTD is critical for RfaH function as it enables recruitment of the 30S ribosomal subunit to mRNAs that lack recognizable Shine-Dalgarno elements, the major component of RfaH activation of gene expression (27). Refolding of the RfaH-CTD into the β-barrel creates the interaction surface for S10 (27), with the resulting RfaH-CTD:S10 complex closely resembling that formed by NusG (28). Why is RfaH autoinhibition so drastic, requiring both the domain dissociation and the CTD refolding? A model where the all-β RfaH-CTD interacts with the NTD as it does in T. maritima NusG (RfaH^βCTD^) reveals a significantly smaller interaction surface than the one in the all-α CTD-inhibited RfaH (Fig. 2B and D; 65). We speculate that a very potent autoinhibition is necessary to tightly control the off-target recruitment of RfaH, which would have severe deleterious effects because RfaH binds to RNAP more tightly than NusG (15). Studies of structures and recruitment of NusG paralogs from other species will reveal their underlying specificity mechanisms.

### NusG-CTD interactions support transcription termination.

Despite its widely accepted role as a transcription processivity factor, E. coli NusG has been long known to promote factor-dependent termination; depletion of NusG compromises termination by Rho and bacteriophage HK202 Nun proteins (78). These termination-promoting activities rely on protein-protein contacts mediated by the NusG-CTD. A recent structure of Rho bound to NusG (32) shows that two loops in the NusG-CTD (Fig. 2A), L1 (residues 140 to 144) and L2 (residues 163 to 167), directly interact with the C terminus of Rho to promote Rho isomerization into an active, translocation-competent state in which the hexameric ring is closed around the nascent RNA (79). An allosteric signal triggered upon NusG binding rearranges a network of intersubunit contacts that maintain Rho in an autoinhibitory state prior to binding to a preferred RNA substrate (32). NusG stimulation is particularly important on sequences that lack high-affinity C-rich Rho loading sites (also known as Rho utilization, or *rut,* sites) and thus represents an important quality control mechanism. Bacterial genomes are pervasively transcribed, generating many nonfunctional RNAs that include antisense and other translation-defective mRNAs. These RNAs would be silenced by Rho but frequently lack canonical *rut* sites. In E. coli, NusG corrects this problem by reprogramming Rho to act on suboptimal C-poor sites (80). While this is an essential function of NusG, at least in E. coli (8), it is not clear how junk RNAs are silenced in other species in which NusG is dispensable, e.g., B. subtilis (81).

In contrast, NusG stimulation of Nun termination appears to be indirect. NusG decreases Nun-mediated transcriptional arrest *in vitro* when present alone, presumably via its antibacktracking activity (17), but potentiates termination/arrest by Nun when NusA, B, and E are also present (82). Substitutions of NusG residues F144 and N145 (in and adjacent to L1) interfere with Nun function (83). Similarly to the wild-type NusG, the F144Y variant decreases RNAP pausing, inhibits Nun arrest, and stimulates Rho but fails to promote Nun-mediated transcription arrest in the presence of other Nus factors (83). This defect is likely explained by weakening of productive interactions with NusE/S10: F144 is located at the interface with NusE in a structure of the NusG:E:B complex (28). This interface is dominated by hydrophobic contacts, which are expected to be weakened by a Tyr substitution.

### Multicomponent complexes that regulate transcription.

**FIG 3:**
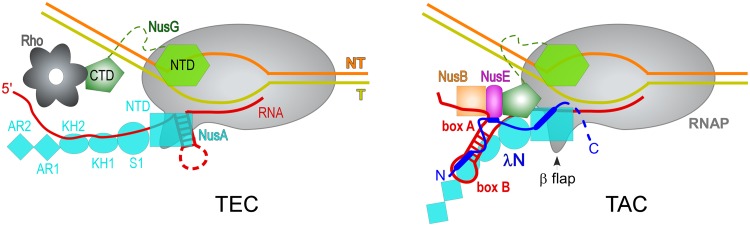
Dual role of NusG in transcription elongation. In the TEC (left), NusG serves as an adapter to enhance the nascent RNA release by Rho, and NusA stabilizes nascent RNA hairpins to stimulate pausing and intrinsic termination. Ongoing NusG-coupled translation (not shown) would protect the RNA from Rho because the same sites on the CTD make direct contacts to Rho and NusE/S10. In the TAC (right), NusA and NusG-CTD interactions are reprogrammed to support the antitermination function of λN. The antibacktracking activity of the NusG-NTD may independently contribute to the pause resistance of the TAC.

Long RNAs that are translated inefficiently are susceptible to premature termination by Rho. Thus, specialized antitermination mechanisms have evolved to protect these transcripts from Rho. Early studies identified NusG as an essential component of multipartite transcription antitermination complexes (TACs) that assemble on nascent RNAs bearing *box A* elements and *box B* hairpins during transcription of phage λ or rRNA genes (84, 85). The shared ability of NusG homologs to decrease RNAP pausing, which is required for termination (86), suggested that NusG contribution to antitermination could be due in part to its antipausing activity. However, a recent medium-resolution cryo-EM structure of a complete λN-dependent TAC (λN-TAC) that contains NusA, NusB, NusE, and NusG and λN paints a picture in which protein-protein contacts take center stage instead (46). λN-TAC is resistant to both hairpin- and Rho-dependent termination (87), but the Nus factors appear to play supporting/stabilizing roles as λN alone has been shown to promote readthrough of intrinsic terminators over short distances (88). An intrinsically disordered λN threads through the TAC and along the RNA, making numerous contacts to RNAP and the Nus factors. Strikingly, λN neutralizes termination-promoting properties of NusA and NusG, converting them into antitermination factors (Fig. 3). λN remodels the β flap domain, which forms one wall of the RNA exit channel, and the RNA-binding domains of NusA to redirect the nascent RNA away from the RNA exit tunnel where formation of a terminator hairpin, stabilized by NusA in the absence of λN (89), would trigger inactivating changes in RNAP (43, 44). These interactions explain how intrinsic termination is disfavored (90) and why Rho may fail to dissociate λN-TAC (91, 92): Rho has to track along the nascent RNA to trigger termination and could thus be sterically blocked from accessing RNAP by NusA domains. The λN-TAC structure also reveals that NusE interacts with L1 and L2 loops of the NusG-CTD, i.e., the same region that binds to Rho (32), thereby preventing NusG activation of Rho through direct exclusion. In addition, their juxtaposition in the complex suggests that NusG-NTD and λN may cooperate to stabilize the upstream edge of the transcription bubble, reducing pausing and termination; NusG and, even more prominently, RfaH display this stabilizing activity (15). A higher-resolution view of λN and NusG interactions with the nucleic acid chains in the λN-TAC would be required to reveal fine details of the antitermination mechanism. In particular, the C-terminal segment of λN, which remained disordered in λN-TAC, could make additional functional interactions.

Using a highly flexible protein hub able to maximize contacts with the TEC and modify its properties emerges as a common strategy in transcription regulation. The termination factor Nun, a functional antipode of λN, is also a small and intrinsically disordered protein. Upon binding to an N-utilization (*nut*) *box A-box B* RNA element (93), Nun arrests the elongating RNAP and blocks its interactions with λN, thus stopping transcription of the λ genome and preventing coinfection with λ (82). A structure of Nun bound to a nonspecific, factorless TEC reveals that Nun sneaks inside the RNAP, making numerous contacts that fill in preexisting cavities in the structure and lock the enzyme in place (45), blocking translocation in either direction (94). In this structure, only a short C-terminal segment of the full-length Nun protein, which is sufficient for Nun-mediated arrest, is visualized. However, just like λN, Nun activity is potentiated by NusA, NusB, NusE, and NusG that assemble on the nascent RNA to bolster its arrest activity (83, 95). Future studies will show whether the missing 80% of Nun binds to and remodels the intrinsically pliable NusA and NusG (or recruits other players) to make a supertermination complex; these contacts could explain observed site-specific differences in Nun action (83).

Another large nucleoprotein complex thought to be assembled from the same cellular building blocks is the ribosomal (*rrn*)-TAC. The *rrn* operons also contain *box A* and *box B* motifs, although in reversed order, and published data show that *rrn*-TAC and λN-TAC share many functional requirements (84, 92). The minimal *rrn*-TAC is composed of Nus factors and RNA, but the identity of a central λN-like hub has remained elusive. The presence of cell extract was shown to stimulate *rrn* antitermination, and ribosomal protein S4 was identified as a key player (96). Other proteins recently implicated in rRNA biosynthesis, SuhB (97) and YbeY (98), may be involved in *rrn* antitermination as well but are more likely to function posttranscriptionally, and the importance of antitermination has been questioned (97). In particular, SuhB plays a critical role in rRNA biogenesis by promoting the maturation of 16S RNA (97). However, while the evidence for S4 contribution to antitermination is solid, it is unclear whether S4 is principally responsible for the potent antitermination activity of the *rrn*-TAC. Secondary RNA structures, stabilized by NusA (or S4), could hinder Rho access (91, 96), but Rho is able to terminate synthesis of highly structured tRNAs (99). Antipausing properties of NusG unmasked by NusE contacts to the NusG-CTD (28) are unlikely to explain Rho inhibition because a much more potent antipausing activity of RfaH (15) is largely dispensable for its anti-Rho effects (12), which are instead due to RfaH activation of translation (27). Perhaps, we should look for an intrinsically disordered protein that can make multidentate contacts with the *rrn*-TAC to hold the complex together and stabilize it. Notably, S4 contains a long flexible N-terminal tail that is essential for viability (100) and could play an analogous role.

### Specialized NusG paralogs.

In addition to the housekeeping NusG/Spt5 present in every cell, specialized paralogs have been identified in many species. NusG^SP^s are broadly distributed in bacteria (64) and are also present in ciliates (101) and plants (102). While functions of most paralogs remain to be determined, the available evidence suggests that they arose via gene duplication and evolved to modulate adaptation to diverse niches ranging from free-living to pathogenic. Most bacteria encode only the housekeeping NusG; in others, several paralogs, as many as seven in Bacteroides fragilis (103), are present. In E. coli, RfaH is encoded on the chromosome, whereas ActX and TraB are encoded on R6K and F plasmids, respectively (104, 105). Bacterial NusG paralogs have been shown to activate biosynthesis of capsules in Klebsiella pneumoniae (106) and B. fragilis (103), toxins in Serratia entomophila (107), antibiotics in Myxococcus xanthus (108) and Bacillus amyloliquefaciens (64), and lipopolysaccharides in several species (14). NusG^SP^s encoded on multidrug-resistant plasmids isolated from clinical K. pneumoniae strains could be essential for the spread of antibiotic-resistant genes, as their location in the pilus biosynthesis operons (109) suggests.

Similar to σ initiation factors, which compete for RNAP core molecules and direct them to dedicated subsets of promoters (110), NusG^SP^s comprise a family of alternative transcription factors that bind to an overlapping site with each other and with σ on elongating RNAP (15, 111). Unlike σs, which all act to direct the formation of active promoter complexes, NusG^SP^s likely function differently from NusG. For example, RfaH activates several long horizontally acquired operons that are silenced by NusG and Rho (11, 12), and the loss of *rfaH* can be suppressed by defects in *rho* and *nusG* (66).

The details of molecular mechanisms by which other NusG^SP^s work are sketchy at best. Their association with long operons, such as 70- to 80-kb antibiotic biosynthesis clusters in M. xanthus (108) and B. amyloliquefaciens (64), is suggestive of a need for specialized antitermination mechanisms. At least in the case of RfaH, inhibition of termination *in vitro* is not potentiated by accessory cellular factors (22), in contrast to *rrn*-TAC (96). One possibility is that, like RfaH, other NusG^SP^s simply lose contacts with Rho and turn into Rho inhibitors. This conversion appears straightforward because key NusG:Rho contacts are highly localized; replacing five residues in its CTD with the corresponding residues of NusG converts RfaH into a potent Rho activator (32). An alternative possibility, suggested by studies of LoaP (64), is that NusG^SP^s could reduce intrinsic hairpin-dependent termination by altering the nascent RNA contacts in the exit channel via CTD:β flap contacts observed with RfaH (15).

### NusG—an adapter between transcription and translation?

In addition to their role in the assembly of TACs and in Rho-dependent termination, contacts between NusG and S10 have been proposed to underpin coupling of transcription and translation in *Bacteria* and *Archaea*, where a nascent RNA emerging from RNAP can be immediately bound by the ribosome (112), protecting it from premature release by Rho (113). Coupling has been observed directly (114), but its timing, mechanism, and extent remain debated (115), and even its existence has been recently called into question (116). Intimate coupling between the two machines is supported by observations that RNAP and the ribosome move in unison (117, 118) and that the lead ribosome blocks RNAP backtracking (118) as well as the formation of the termination hairpin (119). Two modes of coupling have been proposed. In the NusG-coupled model, direct interactions between NusG-CTD and (NusB-bound) S10 captured by NMR spectroscopy (28) link the NusG-NTD-bound RNAP to the ribosome; a short flexible linker that connects the two domains would ensure that the two machines move together yet would allow for some variation in rates. This model is supported by a report that E. coli NusG associates with 70S *in vivo* (29) and by observations that the refolded RfaH-CTD makes similar contacts to S10 (27) and compensates for the lack of Shine-Dalgarno elements on the target mRNA, presumably by recruiting 30S through direct protein-protein contacts. Importantly, while the S10 contacts with NusG and RfaH were first observed with isolated proteins (27, 28), recent structures reveal that these contacts are preserved in the complete E. coli TEC (46, 65). The alternative model posits that RNAP and the ribosome are coupled directly, in the absence of an adapter protein. This model is supported by the cryo-EM structure of an expressome, in which transcribing RNAP establishes multiple interactions with 70S translating the nascent mRNA, leaving essentially no free RNA in between (120), and by direct contacts between RNAP and the ribosome observed in solution (121, 122). Although they appear to be mutually exclusive, both modes of coupling may be utilized on different genes. Analysis of NusG localization within the E. coli genome revealed a significant delay in NusG recruitment to the RNAP transcribing most operons (4), suggesting that coupling, if it occurs on upstream mRNA regions apparently devoid of NusG, is NusG independent.

### Functional cycles of RfaH and NusG.

**FIG 4:**
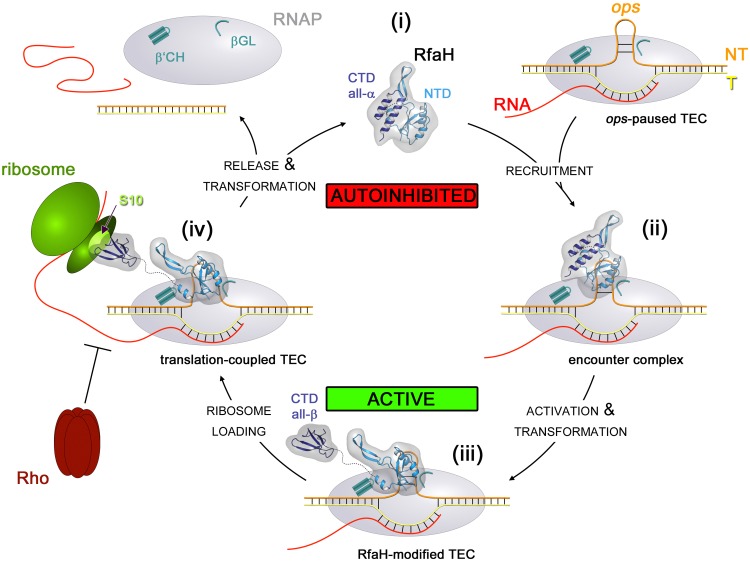
A complete functional cycle of RfaH. (i) Free RfaH exists in a closed, autoinhibited state with its CTD in an α-helical conformation. (ii) Recruitment to the *ops*-paused TEC most probably proceeds via formation of an encounter complex where RfaH makes contacts to the *ops* hairpin and RNAP, positioning RfaH near its final binding site. (iii) The domains dissociate, the RfaH-NTD binds to its high-affinity binding site, and the freed RfaH-CTD is transformed into a NusG-type β-barrel. This active state persists throughout transcription, (iv) hindering Rho-dependent termination and bridging transcription to translation. After transcription stops, the released RfaH transforms back into the autoinhibited state (i).

NusG homolog contact sites on RNAP overlap those for initiation factors, necessitating factor exchange during the transcription cycle (111, 123). This process could be relatively straightforward in the case of NusG, which binds to RNAP relatively weakly and would dissociate during/after termination and then bind again after σ release. An observed delay in NusG recruitment *in vivo* (4) could in some cases be due to persistent σ association (124, 125). RfaH, in contrast, is recruited at promoter-proximal *ops* sites (11), and its off-target recruitment is disfavored by a large excess of NusG (53) and autoinhibition. We posit that during recruitment to an *ops*-paused TEC, autoinhibited RfaH forms a transient encounter complex (Fig. 4) in which the RfaH-NTD can recognize the NT-DNA but is unable to bind to the β′CH until the RfaH-CTD is released. Formation of this complex requires the release of σ and likely involves additional contacts with RNAP, plausibly with the βGL. The functional role and the geometry of the hypothetical encounter complex are yet unknown. Contacts in the encounter complex could (i) increase the local RfaH concentration near the β′CH, facilitating binding of the RfaH-NTD liberated upon transient domain dissociation, or (ii) induce conformational changes that destabilize the interdomain interface. Our failure to observe an exchange between the autoinhibited and activated states of free RfaH by NMR spectroscopy (65) supports the second scenario. Upon domain separation, the RfaH-NTD binds RNAP to form a stable and processive RfaH:TEC complex that persists throughout elongation (11), whereas the RfaH-CTD transforms into the NusG-type β-barrel and binds S10, converting RfaH into a potent activator of translation initiation (27) and possibly linking RNAP and 70S thereafter, as proposed for NusG (29). Finally, we recently showed that upon its dissociation from RNAP RfaH is recycled by transforming back into the autoinhibited state (65), thus resetting the cycle. If RfaH was prematurely released during transcription, recycling would block its reengagement, making the observed RfaH retention on the TEC for thousands of nucleotides (11) even more remarkable. RfaH-CTD contacts with 70S (27) or with RNAP (15) could maintain RfaH in an open, activated state and thus favor its stable association with RNAP. The reversible transition between the autoinhibited and activated states of RfaH bolsters its standing as a “transformer” protein (126). While RfaH plasticity is remarkable even among metamorphic proteins, it is plausible that other members of the NusG family use similar strategies to exert potent, yet exquisitely targeted, effects on gene expression.

### Closing remarks.

Proteins from the NusG family use largely congruent contacts with RNAP to promote productive RNA synthesis but confer very diverse effects on gene expression through interactions with nucleic acids, other regulatory proteins, and potentially small ligands. Studies of just a few representative examples of this family have already documented two different modes of autoinhibition, a complete and reversible refolding of an entire protein domain, a unique mode of DNA recognition with the NT strand serving as a versatile regulatory element, and a novel mechanism of ribosome recruitment. The unprecedented structural plasticity of NusG homologs and the wide range of their interaction partners and resulting activities all but guarantee that future studies will uncover new regulatory mechanisms employed by these ubiquitous proteins.
